# A review on nanoparticles: characteristics, synthesis, applications, and challenges

**DOI:** 10.3389/fmicb.2023.1155622

**Published:** 2023-04-17

**Authors:** Khadijah A. Altammar

**Affiliations:** Department of Biology, College of Science, University of Hafr Al Batin, Hafr Al-Batin, Saudi Arabia

**Keywords:** green synthesis, nanoparticles, nanotechnology, biological synthesis, microbial nanotechnology, bionanotechnology

## Abstract

The significance of nanoparticles (NPs) in technological advancements is due to their adaptable characteristics and enhanced performance over their parent material. They are frequently synthesized by reducing metal ions into uncharged nanoparticles using hazardous reducing agents. However, there have been several initiatives in recent years to create green technology that uses natural resources instead of dangerous chemicals to produce nanoparticles. In green synthesis, biological methods are used for the synthesis of NPs because biological methods are eco-friendly, clean, safe, cost-effective, uncomplicated, and highly productive. Numerous biological organisms, such as bacteria, actinomycetes, fungi, algae, yeast, and plants, are used for the green synthesis of NPs. Additionally, this paper will discuss nanoparticles, including their types, traits, synthesis methods, applications, and prospects.

## 1. Introduction

Nanotechnology evolved as the achievement of science in the 21st century. The synthesis, management, and application of those materials with a size smaller than 100 nm fall under the interdisciplinary umbrella of this field. Nanoparticles have significant applications in different sectors such as the environment, agriculture, food, biotechnology, biomedical, medicines, etc. like; for treatment of waste water (Zahra et al., 2020), environment monitoring (Rassaei et al., 2011), as a functional food additives (Chen et al., 2023), and as a antimicrobial agents (Islam et al., 2022). Cutting-edge properties of NPs such as; nature, biocompatibility, anti-inflammatory and antibacterial activity, effective drug delivery, bioactivity, bioavailability, tumor targeting, and bio-absorption have led to a growth in the biotechnological, and applied microbiological applications of NPs.

A particle of matter with a diameter of one to one hundred nanometers (nm) is commonly referred to as a nanoparticle or ultrafine particle. Nanoparticles frequently exhibit distinctive size-dependent features, mostly due to their tiny size and colossal surface area. The periodic boundary conditions of the crystalline particle are destroyed when the size of a particle approaches the nano-scale with the characteristic length scale close to or smaller than the de Broglie wavelength or the wavelength of light (Guo et al., 2013). Because of this, many of the physical characteristics of nanoparticles differ significantly from those of bulk materials, leading to a wide range of their novel uses (Hasan, 2015).

## 2. Emergence of nanotechnology

Nanotechnology emerged in the 1980s due to the convergence of experimental advances such as the invention of the scanning tunneling microscope in 1981 and the discovery of fullerenes in 1985 (Bayda et al., 2019), with the elucidation. The popularization of a conceptual framework for nanotechnology goals began with the publication of the book Engines of Creation in 1986 (Bayda et al., 2019).

### 2.1. Early stage of NPs

Carbon nanotubes have been discovered in pottery from Keeladi, India, dating from around 600–300 BC (Bayda et al., 2019; Kokarneswaran et al., 2020). Cementite nanowires have been discovered in Damascus steel, a material that dates back to around 900 AD; nevertheless, its origin and creation method are unclear (Kokarneswaran et al., 2020). However, it is unknown how they developed or whether the material containing them was used on purpose.

### 2.2. Discovery of C, Ag, Zn, Cu, and Au nanoparticles

Carbon NPs were found in 1991, and Iijima and Ichihashi announced the single-wall carbon nanotube synthesis with a diameter of 1 nanometer in 1993 (Chen et al., 2021). Carbon nanotubes (CNTs), also known as Bucky tubes, are a kind of nanomaterial made up of a two-dimensional hexagonal lattice of carbon atoms. They are bent one way and joined to produce a hollow cylindrical cylinder. Carbon nanotubes are carbon allotropes that fall between Fullerene (0 dimensional) and Grapheme (2 dimensional) (Chen et al., 2021).

In addition, M. C. Lea reported that the synthesis of citrate-stabilized silver colloid almost 120 years ago (Nowack et al., 2011). This process produces particles with an average diameter of 7 to 9 nm. Nanoscale size and citrate stabilization are analogous to recent findings on nanosilver production employing silver nitrate and citrate (Majeed Khan et al., 2011). The use of proteins to stabilize nanosilver has also been documented as early as 1902 (Nowack et al., 2011; Beyene et al., 2017). Since 1897, a nanosilver known as “Collargol” has been made commercially and used for medicinal purposes (Nowack et al., 2011). Collargol, a type of silver nanoparticle, has a particle size of about 10 nanometers (nm). This was determined as early as 1907, and it was found that the diameter of Collargol falls within the nanoscale range. In 1953, Moudry developed a different type of silver nanoparticle called gelatin-stabilized silver nanoparticles, with a diameter ranging from 2–20 nm. These nanoparticles were produced using another method than Collargol. The necessity of nanoscale silver was recognized by the creators of nanosilver formulations decades ago, as seen by the following remark from a patent: “for optimal efficiency, the silver must be disseminated as particles of colloidal size less than 25 nm in crystallite size”(Nowack et al., 2011).

Gold NPs (AuNPs) have a long history in chemistry, going back to the Roman era when they were used to decorate glassware by staining them. With the work of Michael Faraday, who may have been the first to notice that colloidal gold solutions have characteristics different from bulk gold, the contemporary age of AuNP synthesis began more than 170 years ago. Michael Faraday investigated the making and factors of colloidal suspensions of “Ruby” gold in 1857. They are among the magnetic nanoparticles due to their distinctive optical and electrical characteristics. Under specific illumination circumstances, Faraday showed how gold nanoparticles might create solutions of various colors (Bayda et al., 2019; Giljohann et al., 2020).

## 3. Classification of NPs

Nanoparticles (NPs) are categorized into the following classes based on their shape, size, and chemical characteristics;

### 3.1. Carbon-based NPs

Fullerenes and carbon nanotubes (CNTs) are the two essential sub-categories of carbon-based NPs. NPs of globular hollow cages, like allotropic forms of carbon, are found in fullerenes. Due to their electrical conductivity, high strength, structure, electron affinity, and adaptability, they have sparked significant economic interest. These materials have organized pentagonal and hexagonal carbon units, each of which is sp2 hybridized. While CNTs are elongated and form 1–2 nm diameter tubular structures. These fundamentally resemble graphite sheets rolling on top of one another. Accordingly, they are referred to as single-walled (SWNTs), double-walled (DWNTs), or multi-walled carbon nanotubes (MWNTs) depending on how many walls are present in the rolled sheets (Elliott et al., 2013; Astefanei et al., 2015).

### 3.2. Metal NPs

Metal NPs are purely made of metals. These NPs have distinctive electrical properties due to well-known localized surface Plasmon resonance (LSPR) features. Cu, Ag, and Au nanoparticles exhibit a broad absorption band in the visible region of the solar electromagnetic spectrum. Metal NPs are used in several scientific fields because of their enhanced features like facet, size, and shape-controlled synthesis of metal NPs (Khan et al., 2019).

### 3.3. Ceramics NPs

Ceramic NPs are tiny particles made up of inorganic, non-metallic materials that are heat-treated and cooled in a specific way to give particular properties. They can come in various shapes, including amorphous, polycrystalline, dense, porous, and hollow, and they are known for heat resistance and durable properties. Ceramic NPs are used in various applications, including coating, catalysts, and batteries (Sigmund et al., 2006).

### 3.4. Lipid-based NPs

These NPs are helpful in several biological applications because they include lipid moieties. Lipid NPs typically have a diameter of 10–1,000 nm and are spherical. Lipid NPs, i.e., polymeric NPs, have a solid lipid core and a matrix consisting of soluble lipophilic molecules (Khan et al., 2019).

### 3.5. Semiconductor NPs

Semiconductor NPs have qualities similar to metals and non-metals. That is why Semiconductor NPs have unique physical and chemical properties that make them useful for various applications. For example, semiconductor NPs can absorb and emit light and can be used to make more efficient solar cells or brighter light-emitting diodes (LEDs). They can make smaller and faster electronic devices, such as transistors, and can be used in bio imaging and cancer therapy (Biju et al., 2008).

### 3.6. Polymeric NPs

Polymeric NPs with a size between 1 and 1,000 nm can have active substances surface-adsorbed onto the polymeric core or entrapped inside the polymeric body. These NPs are often organic, and the term polymer nanoparticle (PNP) is commonly used in the literature to refer to them. They resemble Nano spheres or Nano capsules for the most part (Khan et al., 2019; Zielińska et al., 2020).

## 4. Types of different metal-based NPs

Metal NPs are purely made of metal precursors. Due to well-known localized surface plasmon resonance (LSPR) characteristics, these NPs possess unique optoelectrical properties. NPs of the alkali and noble metals, i.e., Cu, Ag, and Au, have a broad absorption band in the visible zone of the solar electromagnetic spectrum. The facet, size, and shape-controlled synthesis of metal NPs are essential in present-day cutting-edge materials (Dreaden et al., 2012; Khan et al., 2019).

### 4.1. Silver nanoparticles (AgNPs)

AgNPs are particles with a size range of 1–100 nanometers made of silver. They have unique physical and chemical properties due to their small size, high surface area-to-volume ratio, and ability to absorb and scatter light in the visible and near-infrared range. Because of their relatively small size and high surface-to-volume ratios, which cause chemical and physical differences in their properties compared to their bulk counterparts, silver nanoparticles may exhibit additional antimicrobial capabilities not exerted by ionic silver (Shenashen et al., 2014).

Besides, AgNPs can be created in various sizes and forms depending on the manufacturing process, the most common of which is chemical reduction. The AgNPs were created by chemically reducing a 12 mM AgNO3 aqueous solution. The reaction was carried out in an argon environment using 70 mL of this solution containing PVP (keeping the molar ratio of the repeating unit of PVP and Ag equal to 34) and 21 mL of Aloe Vera. The mixture was agitated in ultrasonic for 45 min at ambient temperature, then heated 2°C/min to 80°C and left for 2 h to generate a transparent solution with tiny suspended particles that must be removed by simple filtering (Shenashen et al., 2014; Gloria et al., 2017).

### 4.2. Zinc nanoparticles (ZnONPs)

Zinc nanoparticles (ZnONPs) are particles with a size range of 1–100 nm made of zinc. Zinc oxide (ZnO) NPs are a wide band gap semiconductor with a room temperature energy gap of 3.37 eV. Its catalytic, electrical, optoelectronic, and photochemical capabilities have made it widely worthwhile (Kumar S.S. et al., 2013). ZnO nanostructures are ideal for catalytic reaction processes (Chen and Tang, 2007). Laser ablation, hydrothermal methods, electrochemical depositions, sol-gel method, chemical vapor deposition, thermal decomposition, combustion methods, ultrasound, microwave-assisted combustion method, two-step mechanochemical-thermal synthesis, anodization, co-precipitation, electrophoretic deposition, and precipitation processes are some methods for producing ZnO nanoparticles (Madathil et al., 2007; Moghaddam et al., 2009; Ghorbani et al., 2015).

### 4.3. Copper nanoparticles (CuNPs)

Copper nanoparticles (CuNPs) comprise a size range of 1–100 nm of copper-based particles (Khan et al., 2019). Cu and Au metal fluorescence have long been known to exist. For excitation at 488 nm, a fluorescence peak centering on the metals’ interband absorption edge has been noted. Additionally, it was noted that the fluorescence peaked at the same energy at two distinct excitation wavelengths (457.9–514.5 and 300–400 nm), and the high-energy tail somewhat grows with increased photon energy pumping. A unique, physical, top-down EEW approach has been used to create Cu nanoparticles. The EEW method involves sending a current of *1,010 A/m2 (1,010 A/m2) across a thin Cu wire, which explodes on a Cu plate for a duration of 10–6 s (Siwach and Sen, 2008).

### 4.4. Gold nanoparticles (AuNPs)

Gold nanoparticles(AuNPs) are nanometers made of gold. They have unique physical and chemical properties and can absorb and scatter light in the visible and near-infrared range (Rad et al., 2011; Compostella et al., 2017).

Scientists around the turn of the 20th century discovered anisotropic AuNPs. Zsigmond (Li et al., 2014) said that gold particles “are not always spherical when their size is 40 nm or lower” in his book, released in 1909. Additionally, he found anisotropic gold particles of various colors. Zsigmondy won the Nobel Prize in 1925 for “his demonstration of the heterogeneous character of colloidal solutions and the methods he utilized” and for developing the ultramicroscope, which allowed him to see the forms of Au particles. He noticed that gold frequently crystallized into a six-sided leaf shape (Li et al., 2014).

AuNPs are the topic of extensive investigation due to their optical, electrical, and molecular-recognition capabilities, with numerous prospective or promised uses in a wide range of fields, including electron microscopy, electronics, nanotechnology, materials science, and biomedicine (Rad et al., 2011).

### 4.5. Aluminum nanoparticles (AlNPs)

Aluminum nanoparticles (AlNPs) are nanoparticles made of aluminum. Aluminum nanoparticles’ strong reactivity makes them promising for application in high-energy compositions, hydrogen generation in water processes, and the synthesis of alumina 2D and 3D structures (Lerner et al., 2016).

### 4.6. Iron nanoparticles (FeNPs)

Iron nanoparticles(FeNPs) are particles with a size range of 1−100 nanometers (Khan et al., 2019) made of iron. FeNPs have several potential applications, including their use as catalysts, drug delivery systems, sensors, and energy storage and conversion. They have also been investigated for use in photovoltaic and solar cells and water purification and environmental remediation. FeNPs can also be used in magnetic resonance imaging (MRI) as contrast agents to improve the visibility of tissues and organs. They can also be used in magnetic recording media, such as hard disk drives (Zhuang and Gentry, 2011; Jamkhande et al., 2019).

As with any NPs, there are potential health and safety concerns associated with using FeNPs, e.g., FeNPs are used to deliver drugs to specific locations within the body, such as cancer cells and used in MRI, and used to remove contaminants from water (Farrell et al., 2003; Zhuang and Gentry, 2011). Tables 1, 2 show the characteristics of metal-based nanoparticles and the techniques to study their characteristics, respectively.

**TABLE 1:** Characteristics of metal based nanoparticles.

NP	Optimum size	Shape/ Structure	Specific surface area	Aspect ratio	Optical properties	Toxicology	Solubility
AgNPs	1–100 nm (Graf et al., 2003) (Sriram et al., 2012).	Spherical, rod, octagonal, hexagonal, triangle, flower-like (Sriram et al., 2012).	23.81 m^2^/g (Zhou et al., 2009).	For AgNPs synthesized with 40, 80, and 120 mM Fe^3+^ have aspect ratio 490, 1156, and 236, respectively (Saw et al., 2019).	Highly reflective, can be made transparent or translucent (Stepanov et al., 2011).	Low toxicity (Yang et al., 2021).	Excellent water solubility and long-term colloidal stability. (Jana et al., 2007; Rahmati-Abkenar and Manteghian, 2020.)
ZnONPs	1−100 nm (Khan et al., 2019).	Polycrystalline hexagonal structure (Sugihartono et al., 2018).	88.89 m^2^/g (Zhou et al., 2016).	For rod-shaped ZnO nanoparticles is approximay 6 (Wang et al., 2018).	Poorly conductive, it can be made transparent or translucent (Stepanov et al., 2011).	Low toxicity (Yang et al., 2021).	0.3–3.6 mg/L in aqueous medium (Siddiqi and Husen, 2016).
CuNPs	1–100 nm (Khan et al., 2019).	Cubes, rods, tetrahedron, spherically shaped particles (Mott et al., 2007).	5−10 m^2^/g (Ndolomingo and Meijboom, 2016).	For copper nanowires (CuNWs), ranges from 500 to 1666 (Saw et al., 2019)	Highly conductive, can be made transparent or translucent (Stepanov et al., 2011).	Low toxicity (Yang et al., 2021).	Minimal Cu solubility is found at pH 9–11, although above pH 11, CuO solubility increases slightly due to complexing with hydroxide ions (Hortin et al., 2020).
AuNPs	1–100 nm (Khan et al., 2014).	Spherical, triangle, hexagon, and rod (Van Thai et al., 2019).	5.8–107 m^2^/g (Ahmad et al., 2014).	For gold nanorods ranged from 1.83 to 5.04 (Feng et al., 2015).	Highly reflective, gold color (Stepanov et al., 2011).	Low toxicity (Yang et al., 2021)	AuNPs have great solubility in organic solvent such as toluene, while the hydrophilic (1-mercaptoundec-11-yl) tetraethyleneglycol functionalized gold nanoparticles dissolve in water and alcohols (Guo et al., 2015).
FeNPs	1–100 nm (Khan et al., 2019)	Spheres, rods (Essajai et al., 2019; Önal et al., 2019).	14.42 m^2^/g (Essajai et al., 2019; Önal et al., 2019)	–	Poorly conductive, can be made transparent or translucent (Stepanov et al., 2011).	Low toxicity (Yang et al., 2021)	Insoluble in water and inorganic solutions (Essajai et al., 2019)
AlNPs	1–100 nm (Khan et al., 2019)	Nanosphere, nanocubes (Önal et al., 2019)	40–60 m^2^/g (Tavakoli et al., 2013).	–	Poorly conductive, can be made transparent or translucent (Stepanov et al., 2011).	Low toxicity (Yang et al., 2021)	Insoluble in water and soluble in Acetone and ethanol etc, (Wang et al., 2015)

**TABLE 2:** Different analytical techniques and their purposes in studying nanoparticles.

Analytical technique	Purpose	Reference
Centrifugation	To separate the synthesized NPs from reaction solution.	(Patil and Kim, 2018)
Transmission electron microscopy (TEM)	Get High Resolution Pictures than a light microscope. Used to study the structure and presense of NPs.	(Kohl and Reimer, 2008; Patil and Kim, 2018)
Scanning electron microscope (SEM)	Get a three-dimensional appearance 3D based on the interaction of the electron beam with the specimen surface.	(Delvallée et al., 2015)
Scanning tunneling microscopy (STM)	To study the local electronic structure of metal NPs as well as the structure and presence of NPs.	(Wiesendanger and Güntherodt, 2013)
Ultraviolet-visible spectroscopy (UV-Vis)	Used for the optical study of the materials and to determine the synthesis of NPs.	(Patil and Kim, 2018; Rocha et al., 2018)
Fourier transform infrared spectroscopy (FTIR)	To study the surface chemistry of metal NPs. Used for the identification of organic, inorganic, and polymeric materials utilizing infrared light for scanning the samples. Used to identify functional groups in the material.	(Titus et al., 2019; Praseptiangga et al., 2020)
X-ray diffraction (XRD)	Used for characterization of nanopowders of any sizes. Provide useful information and also help correlate microscopic observations with the bulk sample.	(Kahle et al., 2002; Holder and Schaak, 2019)
X-ray photoelectron spectroscopy (XPS)	Used to identify the elemental composition and chemical states of the elements present at the surface of a material.	(Haasch, 2014; Greczynski and Hultman, 2020)
Dynamic light scattering (DLS)	Used to measure the size of particle analyze complex colloidal systems.	(Hoo et al., 2008; Patil and Kim, 2018)
Zeta potential instruments/zeta potential	Measure of the electrical charge at the surface of a particle suspended in a liquid. To study the stability of metal NPs in solution.	(Salopek et al., 1992; Bhattacharjee, 2016)
Small angle X-ray scattering (SAXS)	Used to measure the intensities of X-rays scattered by a sample as a function of the scattering angle.	(Li et al., 2016)
Energy dispersive X-ray spectrometry (EDS), Wavelength dispersive X-ray spectrometry (WDS), X-ray fluorescence spectroscopy (XRF)	Used to identify the elemental composition of a sample.	(Newbury and Ritchie, 2013; Giurlani et al., 2018)
Field emission scanning electron microscope (FESEM)	Used to capture the microstructure image of the materials.	(Lewczuk and Szyryńska, 2021)
Atomic force microscopy (AFM)	Analyze complex colloidal systems obtains information by touching the sample’s surface with a probe used to obtain high-resolution images. To study the size, shape, and surface roughness of metal NPs.	(Cadene et al., 2005; Delvallée et al., 2015)
Particle tracking velocimetry (PTV)	Track individual particles in fluidic systems.	(Kreizer et al., 2010)
Dynamic light scattering (DLS)	Measure the hydrodynamic diameter of nanoparticles in solution.	(De La Calle et al., 2018; Falke and Betzel, 2019)
Nanoparticle tracking analysis (NTA)	Used to obtain the nanoparticle size distribution of samples in liquid suspension. Analyses many particles individually and simultaneously (particle-by-particle).	(Dragovic et al., 2011; Patois et al., 2012)
Raman spectroscopy	Study the vibrational modes of bonds in metal NPs.	(Lyon et al., 1998)
Nuclear magnetic resonance (NMR) spectroscopy	To study the chemical structure and bonding of metal NPs.	(Jayaraman et al., 2014)
Auger electron spectroscopy (AES)	Study the chemical states and bonding of metal NPs.	(Haasch, 2014)
Thermogravimetric analysis (TGA)	Study the thermal stability and decomposition of metal NPs.	(Saldarriaga et al., 2015)
Liquid chromatography	Used to separate and purify compounds that are dissolved in a liquid.	(Chen and Zhu, 2016)

## 5. Approaches for the synthesis of metal NPs

**FIGURE 1:**
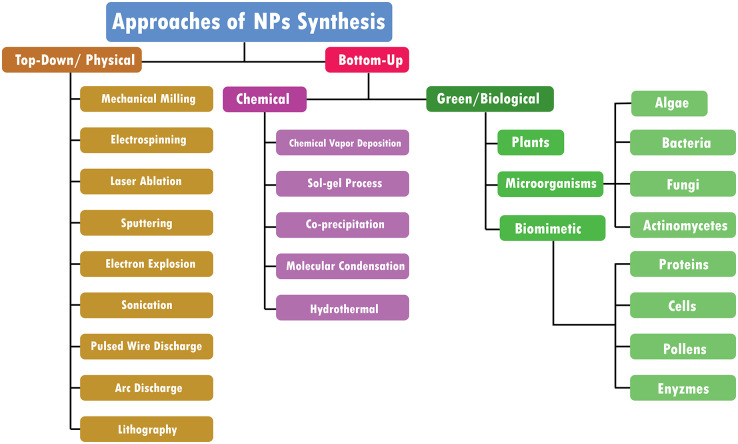
Approaches of NPs synthesis.

There are mainly three types of approaches for the synthesis of NPs: the physical, chemical, and biological approaches. The physical approach is also called the top-down approach, while chemical and biological approaches are collectively called the bottom-up approach. The biological approach is also named green systems of NPs. All these approaches are further sub-categorized into various types based upon their method adopted. Figure 1 illustrates each approach’s reported methods for synthesizing NPs.

### 5.1. Top down/physical approach

**FIGURE 2:**
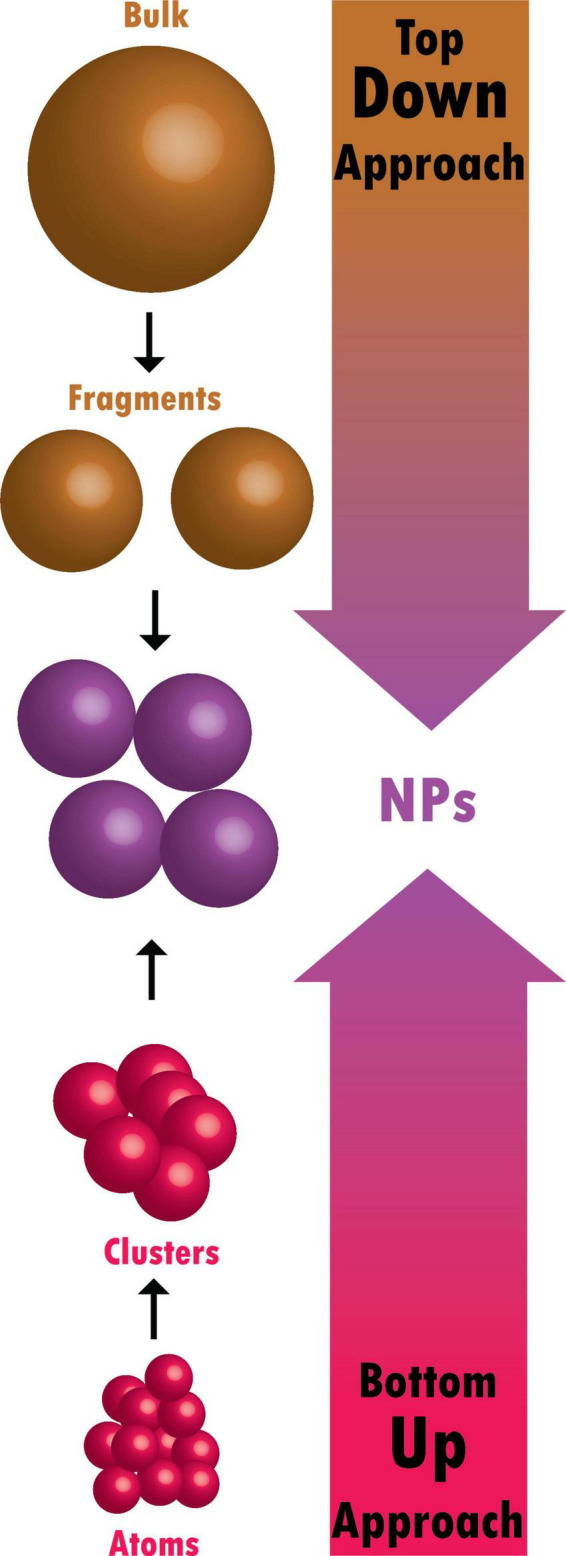
Difference between top-down and bottom-up approaches.

Bulk materials are fragmented in top-down methods to create nano-structured materials (Figure 2). They are additionally known as physical approaches (Baig et al., 2021). The following techniques can achieve a top-down approach;

#### 5.1.1. Mechanical milling

The mechanical milling process uses balls inside containers and may be carried out in various mills, typically planetary and shaker mills, which is an impact process with high energy (Gorrasi and Sorrentino, 2015). Mechanical milling is a practical approach for creating materials at the nanoscale from bulk materials. Aluminum alloys that have been strengthened by oxide and carbide, spray coatings that are resistant to wear, nanoalloys based on aluminum, nickel, magnesium, and copper, and a variety of other nanocomposite materials may all be created mechanically. A unique class of nanoparticles known as ball-milled carbon nanomaterials has the potential to meet the needs for energy storage, energy conversion, and environmental remediation (Yadav et al., 2012; Lyu et al., 2017).

#### 5.1.2. Electrospinning

Typically, it is used to create nanofibers from various materials, most often polymers (Ostermann et al., 2011). A technique for creating fibers called electrospinning draws charged threads from polymer melts or solutions up to fiber sizes of a few hundred nanometers (Chronakis, 2010). Coaxial electrospinning was a significant advancement in the field of electrospinning. The spinneret in coaxial electrospinning is made up of two coaxial capillaries. Core-shell nanoarchitectures may be created in these capillaries using two viscous liquids, a viscous liquid as the shell and a non-viscous liquid as the core (Du et al., 2012). Core-shell and hollow polymer, inorganic, organic, and hybrid materials have all been developed using this technique (Kumar R. et al., 2013).

#### 5.1.3. Laser ablation

A microfeature can be made by employing a laser beam to vaporize a single material (Tran and Wen, 2014). Laser ablation synthesis produces nanoparticles by striking the target material with an intense laser beam. Due to the high intensity of the laser irradiation used in the laser ablation process, the source material or precursor vaporizes, causing the production of nanoparticles (Amendola and Meneghetti, 2009). Laser ablation is an environmentally friendly for producing noble metal nanoparticles (Baig et al., 2021). This method may be used to create a wide variety of nanomaterials, including metal nanoparticles, carbon nanomaterials, oxide composites, and ceramics (Su and Chang, 2018; Baig et al., 2021).

#### 5.1.4. Sputtering

Microparticles of a solid material are expelled off its surface during the phenomenon known as sputtering, which occurs when the solid substance is assaulted by intense plasma or gas particles (Behrisch, 1981). According to the incident gaseous ion energy, energetic gaseous ions used in the sputtering deposition process physically expel tiny atom clusters off the target surface (Muñoz-García et al., 2009). The sputtering method is intriguing because it is more affordable than electron-beam lithography, and the composition of the sputtered nanomaterials is similar to the target material with fewer contaminants (Baig et al., 2021).

#### 5.1.5. Electron explosion

In this technique, a thin metal wire is subjected to a high current pulse that causes an explosion, evaporation, and ionization. The metal becomes vaporized and ionized, expands, and cools by reacting with the nearby gas or liquid medium. The condensed vapor finally forms the nanoparticles (Joh et al., 2013). Electron explosion method because it produces plasma from the electrical explosion of a metallic wire, which may produce nanoparticles from a Pt solution without using a reducing agent (Joh et al., 2013).

#### 5.1.6. Sonication

The most crucial step in the creation of nanofluids is sonication. After the mixture has been magnetically stirred in a magnetic stirrer, sonication is performed in an ultrasonication path, ultrasonic vibrator, and mechanical homogenizer. Sonicators have become the industry standard for Probe sonication and are noticeably more powerful and effective when compared to ultrasonic cleaner baths for nanoparticle applications. Probe sonication is highly effective for processing nanomaterials (carbon nanotubes, graphene, inks, metal oxides, etc.) (Zheng et al., 2010).

#### 5.1.7. Pulsed wire discharge method

This is the most used method for creating metal nanoparticles. A pulsating current causes a metal wire to evaporate, producing a vapor that is subsequently cooled by an ambient gas to form nanoparticles. This plan may quickly produce large amounts of energy (Patil et al., 2021).

#### 5.1.8. Arc discharge method

Two graphite rods are adjusted in a chamber with a constant helium pressure during the Arc Discharge procedure. It is crucial to fill the chamber with helium because oxygen or moisture prevents the synthesis of fullerenes. Arc discharge between the ends of the graphite rods drives the vaporization of carbon rods. Achieving new types of nanoparticles depends significantly on the circumstances in which arc discharge occurs. The creation of several nanostructured materials may be accomplished with this technique (Berkmans et al., 2014). It is well-recognized for creating carbon-based materials such as fullerenes, carbon nanohorns (CNHs), carbon nanotubes (Shi et al., 2000), few-layer graphene, and amorphous spherical carbon nanoparticles (Kumar R. et al., 2013).

#### 5.1.9. Lithography

Lithography typically uses a concentrated beam of light or electrons to create nanoparticles, a helpful technique (Pimpin and Srituravanich, 2012). Masked and maskless lithography are the two primary categories of lithography. Without a mask, arbitrary nano-pattern printing is accomplished in maskless lithography. Additionally, it is affordable and easy to apply (Brady et al., 2019).

### 5.2. Bottom-up approach

Tiny atoms and molecules are combined in bottom-up methods to create nano-structured particles (Figure 2; Baig et al., 2021). These include chemical and biological approaches:

#### 5.2.1. Chemical vapor deposition (CVD)

Through a chemical process involving vapor-phase precursors, a thin coating is created on the substrate surface during CVD (Dikusar et al., 2009). Precursors are deemed appropriate for CVD if they exhibit sufficient volatility, high chemical purity, strong evaporation stability, cheap cost, a non-hazardous nature, and long shelf life. Additionally, its breakdown should not leave behind any contaminants. Vapor phase epitaxy, metal-organic CVD, atomic layer epitaxy, and plasma-enhanced CVD are only a few CVD variations. This method’s benefits include producing very pure nanoparticles that are stiff, homogeneous, and strong (Ago, 2015). CVD is an excellent approach to creating high-quality nanomaterials (Machac et al., 2020). It is also well-known for creating two-dimensional nanoparticles (Baig et al., 2021).

#### 5.2.2. Sol-gel process

A wet-chemical approach, called the sol-gel method, is widely utilized to create nanomaterials (Das and Srivasatava, 2016; Baig et al., 2021). Metal alkoxides or metal precursors in solution are condensed, hydrolyzed, and thermally decomposed. The result is a stable solution or sol. The gel gains greater viscosity as a result of hydrolysis or condensation. The particle size may be seen by adjusting the precursor concentration, temperature, and pH levels. It may take a few days for the solvent to be removed, for Ostwald ripening to occur, and for the phase to change during the mature stage, which is necessary to enable the growth of solid mass. To create nanoparticles, the unstable chemical ingredients are separated. The generated material is environmentally friendly and has many additional benefits thanks to the sol-gel technique (Patil et al., 2021). The uniform quality of the material generated, the low processing temperature, and the method’s ease in producing composites and complicated nanostructures are just a few of the sol-gel technique’s many advantages (Parashar et al., 2020).

#### 5.2.3. Co-precipitation

It is a solvent displacement technique and is a wet chemical procedure. Ethanol, acetone, hexane, and non-solvent polymers are examples of solvents. Polymer phases can be either synthetic or natural. By mixing the polymer solution, fast diffusion of the polymer-solvent into the non-solvent phase of the polymer results. Interfacial stress at two phases results in the formation of nanoparticles (Das and Srivasatava, 2016). This method’s natural ability to produce high quantities of water-soluble nanoparticles through a straightforward process is one of its key benefits. This process is used to create many commercial iron oxide NP-based MRI contrast agents, including Feridex, Reservist, and Combidex (Baig et al., 2021; Patil et al., 2021).

#### 5.2.4. Inert gas condensation/molecular condensation

Metal NPs are produced using this method in large quantities. Making fine NPs using the inactive gas compression approach has been widespread, which creates NPs by causing a metallic source to disappear in an inert gas. At an attainable temperature, metals evaporate at a tolerable pace. Copper metal nanoparticles are created by vaporizing copper metal inside a container containing argon, helium, or neon. The atom quickly loses its energy by cooling the vaporized atom with an inert gas after it boils out. Liquid nitrogen is used to cool the gases, forming nanoparticles in the range of 2–100 nm (Pérez-Tijerina et al., 2008; Patil et al., 2021).

#### 5.2.5. Hydrothermal

In this method, for the production of nanoparticles, hydrothermal synthesis uses a wide temperature range from ambient temperature to extremely high temperatures. Comparing this strategy to physical and biological ones offers several benefits. At higher temperature ranges, the nanomaterials produced by hydrothermal synthesis could become unstable (Banerjee et al., 2008; Patil et al., 2021).

#### 5.2.6. Green/biological synthesis

**FIGURE 3:**
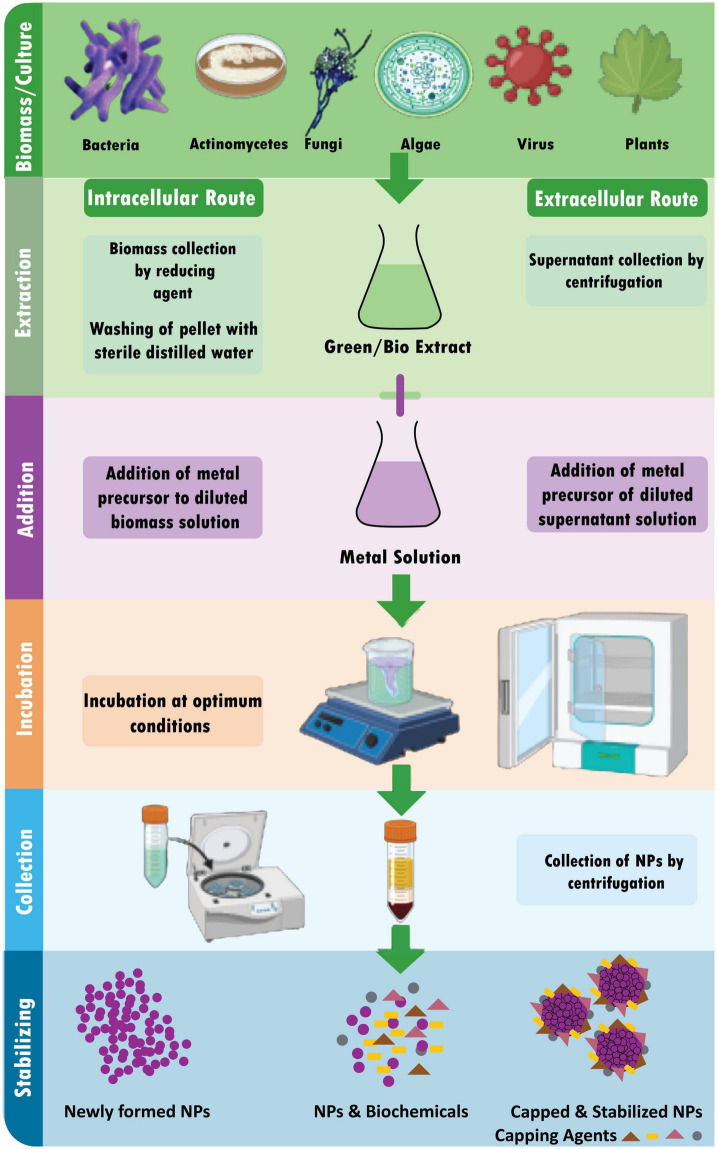
Schematic diagram for biosynthesis of NPs.

The synthesis of diverse metal nanoparticles utilizing bioactive agents, including plant materials, microbes, and various biowastes like vegetable waste, fruit peel waste, eggshell, agricultural waste, algae, and so on, is known as “green” or “biological” nanoparticle synthesis (Kumari et al., 2021). Developing dependable, sustainable green synthesis technologies is necessary to prevent the formation of undesirable or dangerous byproducts (Figure 3). The green synthesis of nanoparticles also has several advantages, including being straightforward, affordable, producing NPs with high stability, requiring little time, producing non-toxic byproducts, and being readily scaled up for large-scale synthesis (Malhotra and Alghuthaymi, 2022).

##### 5.2.6.1. Biological synthesis using microorganisms

Microbes use metal capture, enzymatic reduction, and capping to create nanoparticles. Before being converted to nanoparticles by enzymes, metal ions are initially trapped on the surface or interior of microbial cells (Ghosh et al., 2021). Use of microorganisms (especially marine microbes) for synthesis of metalic NPs is environmental friendly, fast and economical (Patil and Kim, 2018). Several microorganisms are used in the synthesis of metal NPs, including:

Biosynthesis of NPs by bacteria: A possible biofactory for producing gold, silver, and cadmium sulfide nanoparticles is thought to be bacterial cells. It is known that bacteria may produce inorganic compounds either inside or outside of their cells (Hulkoti and Taranath, 2014). *Desulforibrio caledoiensis* (Qi et al., 2013), *Enterococcu*s sp. (Rajeshkumar et al., 2014), *Escherichia coli* VM1 (Maharani et al., 2016), and *Ochrobactrum anhtropi* (Thomas et al., 2014) based metal NPs are reported previously for their potential photocatalytic properties (Qi et al., 2013), antimicrobial activity (Rajeshkumar et al., 2014), and anticancer activity (Maharani et al., 2016).

Extracellular synthesis of NPs by bacteria: The microorganisms’ extracellular reductase enzymes shrink the silver ions to the nanoscale range. According to protein analysis of microorganisms, the NADH-dependent reductase enzyme carries out the bio-reduction of silver ions to AgNPs. The electrons for the reductase enzyme come from NADH, which is subsequently converted to NAD+. The enzyme is also oxidized simultaneously when silver ions are reduced to nanosilver. It has been noted that bio-reduction can occasionally be caused by nitrate-dependent reductase. The decline occurs within a few minutes in the quick extracellular creation of nanoparticles (Mathew et al., 2010). At pH 7, the bacterium *R. capsulata* produced gold nanoparticles with sizes ranging from 10−20 nm. Numerous nanoplates and spherical gold nanoparticles were produced when the pH was changed to four (Sriram et al., 2012). By adjusting the pH, the gold nanoparticles’ form may be changed. Gold nanoparticle shape was controlled by regulating the proton content at various pH levels. The bacteria *R. capsulata*’s release cofactor NADH and NADH-dependent enzymes may cause the bioreduction of Au (3+) to Au (0) and the generation of gold nanoparticles. By using NADH-dependent reductase as an electron carrier, it is possible to start the reduction of gold ions (Sriram et al., 2012).

Intracellular synthesis of NPs by bacteria: Three processes are involved in the intracellular creation of NPs: trapping, bioreduction, and capping. The cell walls of microorganisms and ions charge contribute significantly to creating NPs in the intracellular route. This entails specific ion transit in the presence of enzymes, coenzymes, and other molecules in the microbial cell. Microbes have a range of polysaccharides and proteins in their cell walls, which function as active sites for the binding of metal ions (Slavin et al., 2017). Not all bacteria can produce metal and metal oxide nanoparticles. The only ions that pose a significant hazard to microorganisms are heavy metal ions, which, in response to a threat, cause the germs to react by grabbing or trapping the ions on the cell wall *via* electrostatic interactions. This occurs because a metal ion is drawn to the cell wall’s carboxylate groups, including cysteine and polypeptides, and certain enzymes with a negative charge (Zhang et al., 2011).

Additionally, the electron transfers from NADH *via* NADH-dependent educates, which serves as an electron carrier and is located inside the plasma membrane, causing the trapped ions to be reduced into the elemental atom. The nuclei eventually develop into NPs and build up in the cytoplasm or the pre-plasmic space. On the other hand, the stability of NPs is provided by proteins, peptides, and amino acids found inside cells, including cysteine, tyrosine, and tryptophan (Mohd Yusof et al., 2019).

Biosynthesis of NPs by fungi: Because monodisperse nanoparticles with distinct dimensions, various chemical compositions, and sizes may be produced, the biosynthesis of nanoparticles utilizing fungus is frequently employed. Due to the existence of several enzymes in their cells and the ease of handling, fungi are thought to be great candidates for producing metal and metal sulfide nanoparticles (Mohanpuria et al., 2008).

The nanoparticles were created on the surface of the mycelia. After analyzing the results and noting the solution, it was determined that the Ag + ions are initially trapped on the surface of the fungal cells by an electrostatic interaction between gold ions and negatively charged carboxylate groups, which is facilitated by enzymes that are present in the mycelia’s cell wall. Later, the enzymes in the cell wall reduce the silver ions, causing the development of silver nuclei. These nuclei then increase as more Ag ions are reduced and accumulate on them.

The TEM data demonstrate the presence of some silver nanoparticles both on and inside the cytoplasmic membrane. The findings concluded that the Ag ions that permeate through the cell wall were decreased by enzymes found inside the cytoplasm and on the cytoplasmic membrane. Also possible is the diffusion of some silver nanoparticles over the cell wall and eventual cytoplasmic entrapment (Mukherjee et al., 2001; Hulkoti and Taranath, 2014).

It was observed that the culture’s age does not affect the shape of the synthesized gold nanoparticles. However, the number of particles decreased when older cells were used. The different pH levels produce a variety of shapes of gold nanoparticles, indicating that pH plays a vital role in determining the shape. The incubation temperature also played an essential role in the accumulation of the gold nanoparticles. It was observed that the particle growth rate was faster at increased temperature levels (Mukherjee et al., 2001; Ahmad et al., 2003). The form of the produced gold nanoparticles was shown to be unaffected by the age of the culture. However, when older cells were utilized, the particle count fell. The fact that gold nanoparticles take on various forms at different pH levels suggests that the pH is crucial in determining the shape. The incubation temperature significantly influenced the accumulation of the gold nanoparticles. It was found that higher temperatures caused the particle development rate to accelerate (Mukherjee et al., 2001; Ahmad et al., 2003). *Verticillium luteoalbum* is reported to synthesize gold nanoparticles of 20–40 nm in size (Erasmus et al., 2014). *Aspergillus terreus* and *Penicillium brevicompactum* KCCM 60390 based metal NPs are reported for their antimicrobial (Li G. et al., 2011) and cytotoxic activities (Mishra et al., 2011), respectively.

Biosynthesis of NPs using actinomycetes: Actinomycetes have been categorized as prokaryotes since they share significant traits with fungi. They are sometimes referred to as ray fungi (Mathew et al., 2010). Making NPs from actinomycetes is the same as that of fungi (Sowani et al., 2016). *Thermomonospora* sp., a new species of extremophilic actinomycete, was discovered to produce extracellular, monodispersed, spherical gold nanoparticles with an average size of 8 nm (Narayanan and Sakthivel, 2010). Metal NPs synthesized by *Rhodococcus* sp. (Ahmad et al., 2003) and *Streptomyces* sp. Al-Dhabi-87 (Al-Dhabi et al., 2018) are reported for their antimicrobial activities.

Biosynthesis of NPs using algae: Algae have a high concentration of polymeric molecules, and by reducing them, they may hyper-accumulate heavy metal ions and transform them into malleable forms. Algal extracts typically contain pigments, carbohydrates, proteins, minerals, polyunsaturated fatty acids, and other bioactive compounds like antioxidants that are used as stabilizing/capping and reducing agents (Khanna et al., 2019). NPs also have a faster rate of photosynthesis than their biosynthetic counterparts. Live or dead algae are used as model organisms for the environmentally friendly manufacturing process of bio-nanomaterials, such as metallic NPs (Hasan, 2015). Ag and Au are the most extensively researched noble metals to synthesized NPs by algae either intracellularly or extracellularly (Dahoumane et al., 2017). *Chlorella vulgaris* (Luangpipat et al., 2011), *Chlorella pyrenoidosa* (Eroglu et al., 2013), *Nanochloropsis oculata* (Xia et al., 2013), *Scenedesmus* sp. IMMTCC-25 (Jena et al., 2014) based metal NPs are reported for their potential catalytic (Luangpipat et al., 2011; Eroglu et al., 2013) and, antimicrobial (Eroglu et al., 2013; Jena et al., 2014) activities along with their use in Li-Ion batteries (Xia et al., 2013).

Intracellular synthesis of NPs using algae: In order to create intracellular NPs, algal biomass must first be gathered and thoroughly cleaned with distilled water. After that, the biomass (living algae) is treated with metallic solutions like AgNO3. The combination is then incubated at a specified pH and a specific temperature for a predetermined time. Finally, it is centrifuged and sonicated to produce the extracted stable NPs (Uzair et al., 2020).

Extracellular synthesis of NPs using algae: Algal biomass is first collected and cleaned with distilled water before being used to synthesize NPs extracellularly (Uzair et al., 2020). The following three techniques are frequently utilized for the subsequent procedure:

(i) A particular amount of time is spent drying the algal biomass (dead algae), after which the dried powder is treated with distilled water and filtered.

(ii) The algal biomass is sonicated with distilled water to get a cell-free extract.

(iii) The resultant product is filtered after the algal biomass has been rinsed with distilled water and incubated for a few hours (8–16 h).

##### 5.2.6.2. Biological synthesis using plant extracts

The substance or active ingredient of the desired quality extracted from plant tissue by treatment for a particular purpose is a plant extract (Jadoun et al., 2021). Plant extracts are combined with a metal salt solution at room temperature to create nanoparticles. Within minutes, the response is finished. This method has been used to create nanoparticles of silver, gold, and many other metals (Li X. et al., 2011). Nanoparticles are biosynthesized using a variety of plants. It is known that the kind of plant extract, its concentration, the concentration of the metal salt, the pH, temperature, and the length of contact time all have an impact on how quickly nanoparticles are produced as well as their number and other properties (Mittal and Chisti, 2013). A leaf extract from *Polyalthia longifolia* was used to create silver nanoparticles, the average particle size was around 58 nm (Kumar and Yadav, 2009; Kumar et al., 2016).

*Acacia auriculiformis* (Saini et al., 2016), *Anisomeles indica* (Govindarajan et al., 2016), *Azadirachta indica* (Velusamy et al., 2015), *Bergenia ciliate* (Phull et al., 2016), *Clitoria ternatea*, *Solanum nigrum* (Krithiga et al., 2013), *Coffea arabica* (Dhand et al., 2016), *Coleus forskohlii* (Naraginti et al., 2016), *Curculigo orchioides* (Kayalvizhi et al., 2016), *Digitaria radicosa* (Kalaiyarasu et al., 2016), *Dioscorea alata* (Pugazhendhi et al., 2016), *Diospyros paniculata* (Rao et al., 2016), *Elephantopus scaber* (Kharat and Mendhulkar, 2016), *Emblica officinalis* (Ramesh et al., 2015), *Euphorbia antiquorum* L. (Rajkuberan et al., 2017), *Ficus benghalensis* (Nayak et al., 2016), *Lantana camara* (Ajitha et al., 2015), *Cinnamomum zeylanicum* (Soni and Sonam, 2014), and *Parkia roxburghii* (Paul et al., 2016) are the few examples of plants which are reported for the green synthesis of metal NPs (i.e., AgNPs). These were evaluated for their antifilaria activity (Saini et al., 2016), mosquitocidal activity (Govindarajan et al., 2016), antibacterial activity (Velusamy et al., 2015), catalytic activity (Edison et al., 2016), antioxidant activity (Phull et al., 2016), and Cytotoxicity (Patil et al., 2017).

##### 5.2.6.3. Biological synthesis using biomimetic

“Biomimetic synthesis” typically refers to chemical processes that resemble biological synthesis carried out by living things (Dahoumane et al., 2017). In the biomimetic approach, proteins, enzymes, cells, viruses, pollen, and waste biomass are used to synthesize NPs. Two categories are used to classify biomimetic synthesis:

Functional biomimetic synthesis uses various materials and approaches to emulate particular characteristics of natural materials, structures, and systems (Zan and Wu, 2016).

Process biomimetic synthesis is a technique that aims to create different desirable nanomaterials/structures by imitating the synthesis pathways, processes, or procedures of natural chemicals and materials/structures. For instance, several distinctive nano-superstructures (such as satellite structures, dendrimer-like structures, pyramids, cubes, 2D nanoparticle arrays, 3D AuNP tubes, etc.) have been put together *in vitro* by simulating the protein manufacturing process (Zan and Wu, 2016).

## 6. Applications of NPs

### 6.1. Applications of NPs in environment industry

**FIGURE 4:**
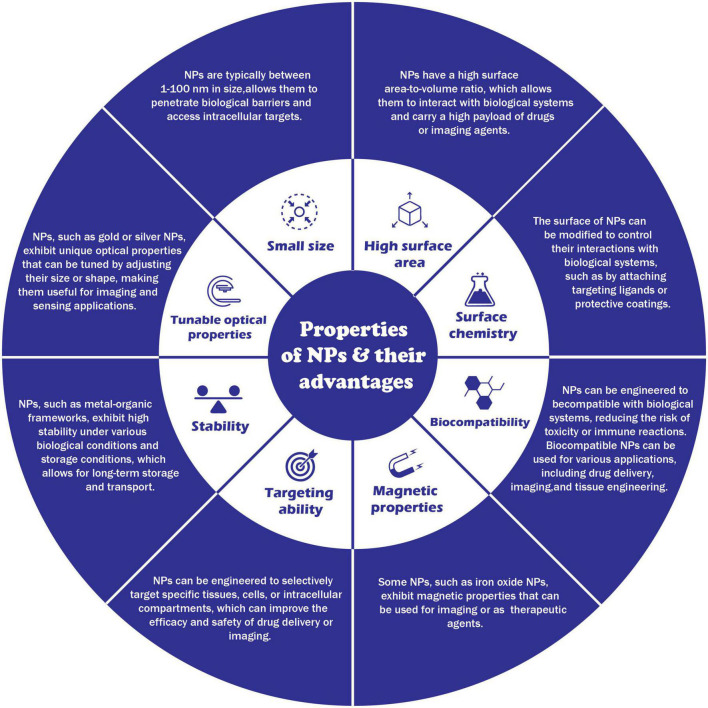
Properties of nanoparticals and their advantages.

Due to their tiny size and distinctive physical and chemical characteristics, NPs appeal to various environmental applications. The properties of nanoparticals and their advantages are illustrated in Figure 4. The following are some possible NP uses in the environment.

#### 6.1.1. Bioremediation

Nanoparticles (NPs) can remove environmental pollutants, such as heavy metals from water or organic contaminants from soil (Zhuang and Gentry, 2011). For example, silver nanoparticles (AgNPs) effectively degrade certain pollutants, such as organic dyes and compounds found in wastewater. Several nanomaterials have been considered for remediation purposes, such as nanoscale zeolites, metal oxides, and carbon nanotubes and fibers (Zhuang and Gentry, 2011). Nanoscale particles used in remediation can access areas that larger particles cannot. They can be coated to facilitate transport and prevent reaction with surrounding soil matrices before reacting with contaminants. One widely used nanomaterial for remediation is Nanoscale zerovalent iron (nZVI). It has been used at several hazardous waste sites to clean up chlorinated solvents that have contaminated groundwater (Elliott et al., 2013). Removing heavy metals such as mercury, lead, thallium, cadmium, and arsenic from natural water has attracted considerable attention because of their adverse effects on environmental and human health. Superparamagnetic iron oxide NPs are an effective sorbent material for this toxic soft material. So, no measurements of engineered NPs in the environment have been available due to the absence of analytical methods able to quantify the trace concentration of NPs (Elliott et al., 2013).

#### 6.1.2. Sensors in environment

Nanotechnology/NPs are already being used to improve water quality and assist in environmental clean-up activities (Pradeep, 2009). Their potential use as environmental sensors to monitor pollutants is also becoming viable NPs can be used as sensors to detect the presence of certain compounds in the environment, such as heavy metals or pollutants. The nano-sensors small size and wide detection range provide great flexibility in practical applications. It has been reported that nanoscale sensors can be used to detect microbial pathogens and biological compounds, such as toxins, in aqueous environments (Yadav et al., 2010). NPS can be designed to selectively bind to specific types of pollutants, allowing them to be detected at low concentrations. For example, gold nanoparticles (AuNPs) have been used as sensors for the detection of mercury in water (Theron et al., 2010).

#### 6.1.3. Catalysts in environment

Nanoparticles (NPs) are used as catalysts in chemical reactions, such as in the production of biofuels or environmental remediation processes, and to catalyze biomass conversion into fuels, such as ethanol or biodiesel. For example, platinum nanoparticles (PtNPs) have been explored for use in the production of biofuels due to their ability to catalyze the conversion of biomass into fuels (Lam and Luong, 2014). PtNPs also showed promising sensing properties; for example, Using Pt NPs, the Hg ions were quantified in the range of 50–500 nM in MilliQ, tap, and groundwater samples, and the limit of quantifications for Hg ions were 16.9, 26, and 47.3 nM. The biogenic PtNPs-based probe proved to be applicable for detecting and quantifying Hg ions (Kora and Rastogi, 2018).

Overall, NPs have significant potential for use in the environment and are being actively researched for a variety of applications.

### 6.2. Applications of NPs in medicine industry

Nanoparticles (NPs) have unique physical and chemical properties due to their small size, making them attractive for use in various applications, including the medicine industry. Some potential applications of NPs in medicine include:

#### 6.2.1. Drug delivery

**FIGURE 5:**
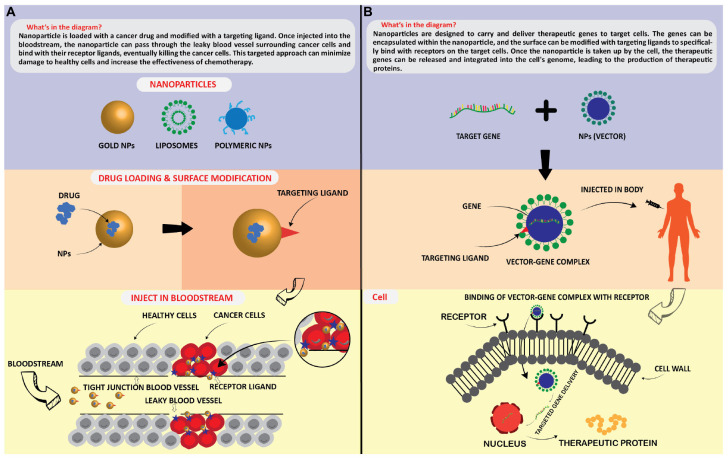
Application of nanoparticles as; targated drug delivery **(A)**, and therapeutic protein generation in targated cells **(B)**.

Technological interest has been given to AuNPs due to their unique optical properties, ease of synthesis, and chemical stability. The particles can be used in biomedical applications such as cancer treatment (Sun et al., 2014), biological imaging (Abdulle and Chow, 2019), chemical sensing, and drug delivery. Sun et al. (2014) mentioned in detail about two different methods of controlled release of drugs associated with NPs, which were (1) sustained (i.e., diffusion-controlled and erosion-controlled) and (2) stimuli-responsive (i.e., pH-sensitive, enzyme-sensitive, thermoresponsive, and photosensitive). Figure 5 illustrates that how NPs acts as targeted delivery of medicines to treat cancer cells (Figure 5A) and therapeutic gene delivery to synthesis proteins of interests in targeted cells (Figure 5B). NPs can deliver drugs to specific body areas, allowing for more targeted and effective treatment (Siddique and Chow, 2020). For example AgNPs have been explored for use in drug delivery due to their stability and ability to accumulate in certain types of cancerous tumors (Siddique and Chow, 2020). ZnONPs have also been explored for drug delivery due to their ability to selectively target cancer cells (Anjum et al., 2021). CuNPs have been shown to have antimicrobial properties and are being explored for drug delivery to treat bacterial infections (Yuan et al., 2018). AuNPs have unique optical, electrical, and catalytic properties and are being explored for drug delivery due to their ability to accumulate in certain cancerous tumors. Silver NPs (AgNPs) have been incorporated into wound dressings, bone cement, and implants (Schröfel et al., 2014).

#### 6.2.2. Diagnostics

Nanoparticles (NPs) can be used as imaging agents to help visualize specific body areas. For example, iron oxide nanoparticles (Fe_3_O_4_ NPs) have been used as magnetic resonance imaging (MRI) contrast agents to help visualize tissues and organs (Nguyen et al., 2013). AuNPs have unique optical, electrical, and catalytic properties and are being explored for diagnostics due to their ability to accumulate in certain cancerous tumors (Siddique and Chow, 2020).

#### 6.2.3. Tissue engineering

Nanoparticles (NPs) can help stimulate the growth and repair of tissues and organs. For example, titanium dioxide nanoparticles (TiO2 NPs) have been explored for tissue engineering due to their ability to stimulate the growth of bone cells (Kim et al., 2014).

#### 6.2.4. Antimicrobials

Some NPs, such as silver nanoparticles (AgNPs) and copper nanoparticles (CuNPs), have strong antimicrobial properties and are being explored for use in a variety of medical products, such as wound dressings and medical devices (Hoseinzadeh et al., 2017).

Overall, NPs have significant potential for use in the medical industry and are being actively researched for various applications. However, it is essential to carefully consider the potential risks and benefits of using NPs in medicine and ensure their safe and responsible use.

### 6.3. Applications of NPs in agriculture industry

There are several ways in which nanoparticles (NPs) have the potential to alter the agricultural sector. NPs may be used in agriculture for a variety of reasons, including:

#### 6.3.1. Pesticides and herbicides

Nanoparticles (NPs) can be used to deliver pesticides and herbicides in a targeted manner, reducing the number of chemicals needed and minimizing the potential for environmental contamination (Khan et al., 2019). AgNPs and CuNPs have antimicrobial properties, making them potentially useful for controlling pests and diseases in crops. They can also be used as delivery systems for active ingredients, allowing for more targeted application and reducing the potential for environmental contamination (Hoseinzadeh et al., 2017; Dangi and Verma, 2021).

It is important to note that using metal NPs in pesticides and herbicides is still in the early stages of development. More research is needed to understand their potential impacts on human health and the environment (Dangi and Verma, 2021).

#### 6.3.2. Fertilizers and plant growth

Nano fertilizers offer an opportunity for efficiently improving plant mineral nutrition. Some studies have shown that nanomaterials can be more effective than conventional fertilizers, with a controlled release of nutrients increasing the efficiency of plant uptake and potentially reducing adverse environmental outcomes associated with the loss of nutrients in the broader environment. However, other studies have found that nanomaterial has the same or even less effective effectiveness than conventional fertilizers. NPs used to deliver fertilizers to plants more efficiently, reducing the amount of fertilizer needed, and reducing the risk of nutrient runoff (Kopittke et al., 2019).

Ag (Jaskulski et al., 2022), Zn (Song and Kim, 2020), Cu, Au, Al, and Fe (Kopittke et al., 2019) based NPs have been shown to have fertilizing properties and plant growth-promoting properties, and may help provide essential nutrients to plants and improve plant growth and yield. It is important to note that the use of NPs in fertilizers is still in the early stages of development. More research is needed to understand their potential impacts on human health and the environment.

#### 6.3.3. Food safety

Nanoparticles (NPs) can detect and eliminate pathogens in food products, improving food safety, and reducing the risk of foodborne illness (Zhuang and Gentry, 2011).

#### 6.3.4. Water purification

Nanoparticles (NPs) can purify irrigation water, reducing the risk of crop contamination and improving crop yield (Zhuang and Gentry, 2011). Using NPs in agriculture can improve crop yields, reduce agriculture’s environmental impact, and improve food products’ safety and quality.

### 6.4. Applications of NPs in food industry

Numerous applications for nanoparticles (NPs) in the food sector are possible, including:

#### 6.4.1. Food processing and food preservation/food packaging

Nanoparticles (NPs) can be used to improve the efficiency and performance of food processing operations, such as grinding, mixing, and drying, e.g., AgNPs have been used as a natural antimicrobial agent in food processing operations, helping to prevent the growth of bacteria and other microorganisms (Dangi and Verma, 2021) and also NPs are used to enhance the performance of materials used in food packaging, making them more resistant to pollutants like moisture and gases.

#### 6.4.2. Food fortification

Nanoparticles (NPs) can deliver essential nutrients to food products, such as vitamins and minerals, more efficiently and effectively. e.g., Fe_2_O_3_, and CuNPs have been used to fortify food products with iron, and Cu is an essential nutrient necessary for the metabolism of iron and other nutrients. Iron is an essential nutrient often lacking in many people’s diets, particularly in developing countries (Kopittke et al., 2019).

#### 6.4.3. Sensors

Nanoparticles (NPs) used to improve the sensitivity and specificity of food sensors, allowing them to detect a broader range of substances or signals (Yadav et al., 2010).

Overall, using NPs in the food industry can improve the performance, safety, and nutritional value of a wide range of food products and processes.

### 6.5. Applications of NPs in electronics industry and automotive industry

In many aspects, nanoparticles (NPs) can transform the electronics sector. NPs may be used in a variety of electrical applications, such as:

#### 6.5.1. Display technologies/storage devices

Nanoparticles (NPs) can be used to improve the performance of displays (Park and Choi, 2019; Bahadur et al., 2021; Triana et al., 2022), such as LCD and OLED displays, by enhancing the brightness, color, and contrast of the image, such as silver NPs and gold NPs, have been explored for use in LCD and OLED displays as a means of improving the conductivity of the display (Gwynne, 2020). NPs improve the performance and durability of energy storage devices, such as batteries and supercapacitors, by increasing energy density and charging speed. Zinc oxide nanoparticles (ZnO NPs) have the potential to be used in energy storage devices, such as batteries and supercapacitors, due to their ability to store and release energy (Singh et al., 2011).

#### 6.5.2. Data storage

Nanoparticles (NPs) can improve the capacity and speed of data storage devices, such as hard drives and flash drives. Magnetic NPs, such as iron oxide NPs, have been explored for use in data storage devices, such as hard drives, due to their ability to store, and retrieve data using magnetism. These NPs are often composed of a magnetic metal, such as iron, cobalt, or nickel. They can be magnetized and demagnetized, allowing them to store and retrieve data (Ahmad et al., 2021).

Overall, the use of NPs in electronics has the potential to improve the performance and efficiency of a wide range of electronic devices and systems.

Applications of NPs in chemical industry: The chemical industry might be entirely transformed by nanoparticles (NPs) in various ways. The following are potential uses for NPs in the chemical industry (Salem and Fouda, 2021).

#### 6.5.3. Chemical processing/catalysis

Nanoparticles (NPs) can be used as catalysts in chemical reactions, allowing them to be carried out more efficiently and at lower temperatures. Some examples of metal NPs that have been used as catalysts in the chemical industry include: PtNPs have been used as catalysts in a variety of chemical reactions, including fuel cell reactions (Bhavani et al., 2021), hydrogenation reactions, and oxidation reactions (Lara and Philippot, 2014), PdNPs have been used as catalysts in a variety of chemical reactions, including hydrogenation reactions and cross-coupling reactions (Pérez-Lorenzo, 2012), FeNPs have been used as catalysts in a variety of chemical reactions, including hydrolysis reactions (Jiang and Xu, 2011), and oxygen reduction reactions, NiNPs have been used as catalysts in a variety of chemical reactions, including hydrogenation reactions, and hydrolysis reactions (Salem and Fouda, 2021).

#### 6.5.4. Separation and purification

NPs are used to separate and purify chemicals and other substances, such as gases and liquids, by exploiting their size-based properties (Hollamby et al., 2010). Several types of metal nanoparticles (NPs) have been explored for use in separation and purification processes in the chemical industry, including Fe_2_O_3_ NPs have been used to separate and purify gases, liquids, and chemicals. They have also been used to remove contaminants from water (Pradeep, 2009; Siddique and Chow, 2020). AgNPs have been used to purify water and remove contaminants (Pradeep, 2009), such as bacteria and viruses. They have also been used to remove heavy metals from water and other substances (Zhuang and Gentry, 2011). AuNPs have been used to purify water and remove contaminants, such as bacteria and viruses (Siddique and Chow, 2020). They have also been used to separate and purify gases and liquids (Zhuang and Gentry, 2011). AlNPs have been used to remove contaminants from water and other substances, such as oils and fuels. They have also been used to purify gases (Zhuang and Gentry, 2011).

### 6.6. Applications of NPs in defense industry

Nanoparticles (NPs) can be used to improve the efficiency and performance of chemical processing operations, such as refining and synthesizing chemicals (Schröfel et al., 2014). Nanoparticles (NPs) have the potential to be used in the defense industry in several ways, including:

#### 6.6.1. Sensors

Nanoparticles (NPs) can improve the sensitivity and specificity of sensors used in defense systems, such as sensors for detecting chemical, biological, or radiological threats (Zheng et al., 2010).

#### 6.6.2. Protective coatings

Nanoparticles (NPs) can improve the performance and durability of protective coatings applied to defense equipment, such as coatings resistant to chemical or biological agents. For example, metal NPs can improve the mechanical properties and durability of the coating, making it more resistant to wear and corrosion. For example, adding Al or Zn based NPs to a polymer coating can improve its corrosion resistance. In contrast, adding Ni or Cr-based NPs can improve their wear resistance (Rangel-Olivares et al., 2021).

#### 6.6.3. Weapons

Nanoparticles (NPs) are used as weapons against viruses, bacteria, etc, (Ye et al., 2020) and as well as in the development of armor and protective materials. There have been some reports of the potential use of NPs in military and defense applications, such as in the development of armor and protective materials. For example, adding nanoparticles, such as ceramic or metal NPs, to polymers or other materials can improve their mechanical properties and make them more resistant to damage. In addition, there have been reports of the use of NPs in developing sensors and detection systems for defense purposes.

#### 6.6.4. Manufacturing

Nanoparticles (NPs) can improve the performance and durability of materials used in defense equipment, such as armor or structural materials. Metal NPs can be used in materials by adding them as a filler or reinforcement in polymers. For example, the addition of metal NPs such as aluminum (Al), copper (Cu), or nickel (Ni) to polymers can improve the mechanical properties, thermal stability, and electrical conductivity of the resulting composite material (Khan et al., 2019).

Metal NPs can also make functional materials, such as catalysts and sensors. For example, metal NPs, such as gold (Au), and platinum (Pt), can be used as catalysts in various chemical reactions due to their high surface area and ability to adsorb reactants (Zheng et al., 2010).

#### 6.6.5. Energy storage

Nanoparticles (NPs) can improve the performance and efficiency of energy storage systems used in defense systems, such as batteries or fuel cells (Morsi et al., 2022). In batteries, nanoparticles can be used as a cathode material to increase the battery’s energy density, rate capability, and cycling stability. For example, lithium cobalt oxide (LiCoO_2_) nanoparticles have been used as cathode materials in lithium-ion batteries due to their high capacity and good rate performance. In addition, nanoparticles of transition metal oxides, such as iron oxide (Fe_2_O_3_), and manganese oxide (MnO_2_), have been used as cathode materials in rechargeable lithium batteries due to their high capacity and good rate performance. In supercapacitors, nanoparticles can be used as the active material in the electrodes to increase the specific surface area, leading to an increase in the device’s capacitance (Morsi et al., 2022). Using NPs in the defense industry can improve defense systems’ performance, efficiency, and safety.

## 7. Future perspectives

Metal nanoparticles (NPs) have many potential applications in various fields, including electronics, energy storage, catalysis, and medicine. However, there are also several challenges and potential future directions for developing and using metal NPs.

One major challenge is synthesizing and processing metal NPs with precise size and shape control. Many methods for synthesizing metal NPs involve high temperatures and harsh chemical conditions, which can be challenging to scale up for large-scale production. In addition, the size and shape of metal NPs can significantly impact their properties and potential applications, so it is essential to synthesize NPs with precise size and shape control.

Another challenge is the environmental impact of metal NPs. Some metal NPs, such as silver NPs, can be toxic to aquatic life and may have other environmental impacts. There is a need for more research on the environmental effects of metal NPs and the development of more environmentally friendly (Green) synthesis and processing methods.

In terms of future directions, one promising area is the use of metal NPs for energy storage, conversion, and protection of the environment. For example, metal NPs could be used to improve batteries’ performance or develop more efficient solar cells. In addition, metal NPs could be used in catalysis to improve the efficiency of chemical reactions. There is also ongoing research on metal NPs in medicine, including drug delivery and cancer therapy.

## Author contributions

KAA: conceptualization, methodology, validation, formal analysis, investigation, writing – original draft, writing – review and editing, and visualization.
